# A Microfluidic Co‐Flow Route for Human Serum Albumin‐Drug–Nanoparticle Assembly

**DOI:** 10.1002/chem.202001146

**Published:** 2020-04-28

**Authors:** Tuuli A. Hakala, Sarah Davies, Zenon Toprakcioglu, Barbara Bernardim, Gonçalo J. L. Bernardes, Tuomas P. J. Knowles

**Affiliations:** ^1^ Department of Chemistry University of Cambridge Lensfield Road CB2 1EW Cambridge UK; ^2^ Instituto de Medicina Molecular Faculdade de Medicina Universidade de Lisboa Avenida Professor Egas Moniz 1649-028 Lisboa Portugal

**Keywords:** albumin, celastrol, drug delivery, microfluidics, nanoparticles

## Abstract

Nanoparticles are widely studied as carrier vehicles in biological systems because their size readily allows access through cellular membranes. Moreover, they have the potential to carry cargo molecules and as such, these factors make them especially attractive for intravenous drug delivery purposes. Interest in protein‐based nanoparticles has recently gained attraction due to particle biocompatibility and lack of toxicity. However, the production of homogeneous protein nanoparticles with high encapsulation efficiencies, without the need for additional cross‐linking or further engineering of the molecule, remains challenging. Herein, we present a microfluidic 3D co‐flow device to generate human serum albumin/celastrol nanoparticles by co‐flowing an aqueous protein solution with celastrol in ethanol. This microscale co‐flow method resulted in the formation of nanoparticles with a homogeneous size distribution and an average size, which could be tuned from ≈100 nm to 1 μm by modulating the flow rates used. We show that the high stability of the particles stems from the covalent cross‐linking of the naturally present cysteine residues within the particles formed during the assembly step. By choosing optimal flow rates during synthesis an encapsulation efficiency of 75±24 % was achieved. Finally, we show that this approach achieves significantly enhanced solubility of celastrol in the aqueous phase and, crucially, reduced cellular toxicity.

## Graphical abstract

**Nanotechnology**: We demonstrate a microfluidic co‐flow device to produce drug‐loaded albumin nanoparticles without additional cross‐linking steps. This method showed high loading efficiency of the lipophilic drug celastrol, which created spherical nanoparticles with considerable reduced drug cytotoxicity (see figure).

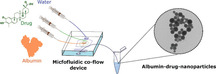

Targeted delivery and controllable release of active pharmaceuticals are major objectives to improve the safety and efficacy of potential drugs. These important properties can be enhanced by using suitable drug carriers, such as nanoparticles. Nanoparticles are an attractive class of carrier in this context because they can solubilize therapeutic cargo, which can prolong the circulation lifetimes of drugs and ability to extravasate to tumour sites.^1^ These therapeutic cargo carriers need to be very biocompatible to decrease the risk of unwanted complications. Thus, protein nanoparticles, which intrinsically have minimal immunogenicity and biocompatibility, have attracted a lot of interest.^2^ An additional benefit of using proteins for nanoparticle formation is that they can be selectively modified with specific ligands for targeting purposes.^3^ Previously, proteins have been applied to increase stability in microdroplets^4^ and form protein‐based nanoparticles from bovine serum albumin (BSA),^5^ human serum albumin (HSA)^6^ and β‐lactoglobulin.^7^ Interestingly, albumin nanoparticles have been shown to penetrate through the blood–brain barrier,^8^ which broadens the areas where therapeutic agents could be delivered.

There are a wide variety of methods available for nanoparticle formation including nano emulsification and spray drying.^9^ However, these methods require chemical cross‐linking or proteins to form fibrillar networks. Another, popular method for nanoparticle formation is desolvation, in which a protein or polymer is in aqueous media and a desolvating agent, such as ethanol, is added drop by drop.^10^ In this method the introduction of the desolvating agent to the protein solution, de‐stabilises the protein structure and exposes its buried hydrophobic and reactive residues. This promotes interactions between protein molecules, so that the proteins clump together in small aggregate nanoparticles.^11^ Recently, a method to introduce intermolecular disulfide bonds between HSA molecules to avoid the use of toxic crosslinkers was reported.^12^ This approach relied on an additional denaturing step prior to nanoparticle formation, which reduced some of the HSA's disulfide bridges and further promoted cysteine‐cysteine interactions between different HSA molecules during nanoparticle formation.

Herein, we present a microfluidic co‐flow strategy for nanoparticle formation without the need for additional cross‐linkers. This co‐flow method is based on the desolvation method, but instead of dropwise addition, the desolvating agent flows adjacent to the protein solution in a microfluidic chip. In this approach, mixing is much faster with no gradual increase of the desolvating agent concentration. Furthermore, we encapsulated a highly lipophilic drug, celastrol, into HSA nanoparticles. Celastrol is a natural compound extracted from herb *Tripterygium wilfordii*, a direct modulator of progesterone and cannabinoid receptors^13^ that elicits a potent anti‐inflammatory response^14^ and shows promise as a treatment for Alzheimer's disease,^14^ obesity,^15^ rheumatoid arthritis^16^ and cancer.^17^ Due to its hydrophobicity, celastrol is difficult to use in aqueous solutions. Therefore, it would be a significant advance if this molecule could be encapsulated into a more hydrophilic shell. With our approach, we show encapsulation of celastrol with high efficiency within HSA nanoparticles, which increases celastrol's solubility and simultaneously dramatically decreases its cytotoxicity.

To showcase the variety of this co‐flow method to produce nanoparticles, we initially investigated the formation of BSA nanoparticles by using ethanol as the desolvating agent. BSA was introduced from the middle inlet and surrounded by ethanol (Figure 1 a and **1 b**). Additionally, a water stream was added as the outer layer. This water layer has two purposes: first, the water pinches the ethanol and protein stream so that there will be fast diffusion between the streams. Secondly, the water layer will ensure that the protein does not come in contact with the hydrophobic polydimethylsiloxane (PDMS) walls to avoid surface adherence. To completely envelop the protein and ethanol streams with water a 3D co‐flow design was used (Figure 1 c). In a conventional 2D microfluidic chip the fluid streams flow adjacent to one another, whereas in 3D devices one stream flows within the another (Figure 1 c).

**Figure 1:**
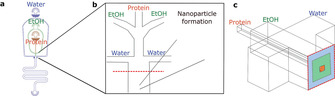
Microfluidic co‐flow device design: Schematic representation of the microfluidic co‐flow method for synthesis of protein nanoparticles. (a) CAD design from the co‐flow device, in which water flows from the outer channel, ethanol from the middle channel, and protein from the inner channel. (b) The three solutions meet in the middle of the device to form the nanoparticle. (c) The 3D channel geometry gives co‐flow layers.

Our 3D co‐flow device was applied to study BSA nanoparticle formation with two different concentrations and six different flow rates. Figure 2 shows the size distributions measured with dynamic light scattering (DLS; Figures 2 a and **2 d**) and average diameters (Figures 2 b and **2 e**) from BSA nanoparticles prepared from 1 mg mL^−1^ (Figures 2 a and **2 b**) and 10 mg mL^−1^ (Figures 2 d and **2 e**) BSA solutions. This study revealed that whilst the concentration has only a limited effect on the average size, it has a larger effect on the size distributions. Furthermore, polydispersity in the nanoparticle samples produced with a 10 mg mL^−1^ BSA solution was larger, showing an increase in recorded polydispersity indexes (PDI; Table S1). This is also evident from the DLS size distributions for which broader peaks are observed (Figure 2 d). Furthermore, larger standard deviations were recorded for the average diameters (Figure 2 e). A more profound effect was achieved by changing the EtOH to protein flow‐rate ratio. The higher the EtOH flow rate, the higher the concentration of EtOH in the resulting mixture, and subsequently the bigger the nanoparticles formed. With 1 mg mL^−1^ BSA solution the average size increases exponentially. However, in the case of 10 mg mL^−1^ BSA solution, the increase in average diameter is more linear. This can be explained by the 10‐fold increase of protein molecules as the EtOH concentration remains constant. Thus, the curve is shifted to the right as BSA concentration is increased. The morphology of BSA nanoparticles was examined by transmission electron microscopy (TEM). Figure 2 shows BSA nanoparticles made from 1 mg mL^−1^ (Figure 2 c parts **i** and **ii**) and 10 mg mL^−1^ solutions (Figure 2 f parts **i** and **ii**), using a 1:1 flow ratio. The particles were highly spherical for both protein concentrations. However, based on the TEM images the polydispersity seems to be larger in the case of nanoparticles made from 10 mg mL^−1^ solution, which confirms the results obtained from DLS.

**Figure 2:**
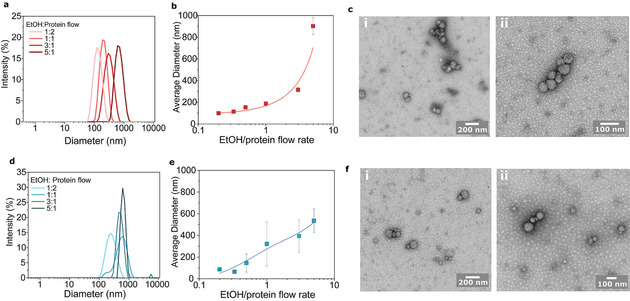
BSA nanoparticle characterisation: Protein nanoparticle formation was characterized using two different BSA concentrations. (a–c) 1 mg mL^−1^ and (d–f) 10 mg mL^−1^ solutions were used and characterised with (a, b, d and e) DLS and TEM (c and f). Size distributions (a and d) and average size (b and e) were recorded for six different flow ratios (ethanol/protein flow rate). And TEM images were taken for (c i and ii) 1 mg and (f i and ii) 10 mg mL^−1^ BSA nanoparticles with 1:1 flow ratio.

To determine whether the BSA nanoparticles remain stable, the zeta potential for the two different solutions was measured in phosphate‐buffered saline (PBS; pH 7.3) for particles created with 1:1 flow rate ratio (Figure 3 a). Generally, zeta potential values that are not in the range −30±30 mV are generally considered to have sufficient repulsive force to attain better physical colloidal stability.^18^ From the data obtained, both samples have a relatively high zeta potential (−46.1 for 1 mg mL^−1^ and −29.2 for 10 mg mL^−1^) and are stable in solution. To further elucidate our understanding of the nanoparticle formation, an 8‐anilinonaphthalene‐1‐sulfonic acid (ANS) assay was conducted. ANS binds to hydrophobic cavities found on the protein surface and increases its fluorescence intensity upon binding.^19^ Thus, the more hydrophobic residues are exposed on the sample, the higher the fluorescence signal. In Figure 3 b the fluorescence signal of a 3 μm BSA solution and a 3 μm nanoparticle based BSA solution is shown. The ANS fluorescence showed decreased signal with BSA nanoparticles, which indicates that the hydrophobic pockets are less exposed in the nanoparticles compared to native BSA. This suggests that the interactions between hydrophobic residues are driving protein nanoparticle formation.

**Figure 3:**
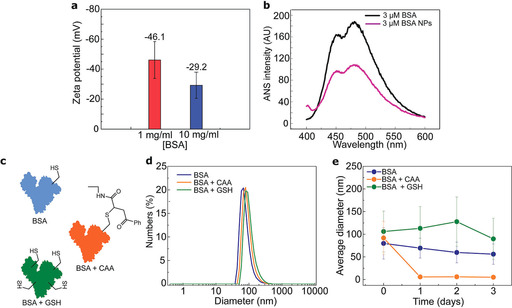
Stability of BSA nanoparticles: (a) Zeta potential measurement for nanoparticles made from 1 and 10 mg mL^−1^ BSA solutions by using a 1:1 flow ratio (ethanol/water). (b) ANS binding to free BSA and BSA nanoparticles. (c) Stability in aqueous solution (PBS, pH 7.3) was examined with three different samples: native BSA (BSA, blue), BSA with blocked free cysteine (BSA+CAA, orange) and GSH reduced BSA (BSA+GSH, green). (d) Size distributions for these three samples following their formation by using the co‐flow method and (e) average diameter after 1,2 and 3 days of incubation in 23±2 °C. The error bars in panel e represent the standard deviations of the size distributions.

Typically, when forming protein nanoparticles, the use of toxic crosslinkers is employed to stabilize the system. However, such systems can have adverse health effects and these crosslinked nanoparticles cannot always be used safely for in vivo delivery applications. Here, the protein was reduced with glutathione (GSH) to increase the number of free cysteines and promote the formation of intermolecular disulfide bridges.^12^ To prove the involvement of disulfide bridges in the nanoparticle stability, three different protein solutions were prepared: BSA, BSA reduced with glutathione (GSH) and BSA in which the free cysteine was blocked with a cysteine selective carbonylacrylic linker (CAA).^20^ Total conversion from free cysteine to blocked cysteine by using a CAA linker was observed (see Figures S3 and S4). All three solutions were used to prepare BSA nanoparticles and the stability was measured over time in PBS solution (pH 7.3). Initially, the size distributions were quite similar, as determined by DLS (Figure 3 d). However, the differences in stability over time are evident (Figure 3 e). Both BSA and reduced BSA remained as nanoparticles throughout the duration of the experiment. However, as expected the nanoparticles in which the formation of disulfide bonds was blocked disassembled within 24 h. Furthermore, nanoparticles made from native BSA showed a decreasing linear trend in average size during the stability measurement. This indicates lower stability relative to nanoparticles made from reduced BSA.

Following the characterisation of nanoparticles formed by using the co‐flow, a protein‐drug particle was established, with potential for clinical application. We chose to use HSA in this study since it is the most abundant protein in human plasma and is therefore widely used for drug delivery.^21^ Moreover, due to its structural similarity to BSA, it could be assumed to work similarly to BSA in the co‐flow method. Celastrol was chosen as a cargo molecule due to its potential as a therapeutic molecule.^13^ However, its use is still limited due to its highly lipophilic nature and cytotoxicity. Thus, it would be of significant relevance if this molecule could be encapsulated into a more hydrophilic and less toxic shell. HSA/celastrol nanoparticles were created by using the previously established strategy by co‐flowing HSA with celastrol in EtOH (Figure 4 a). Similarly, to BSA, HSA was partly reduced prior to microfluidic co‐flow to increase the number of intermolecular disulfide bridges and further to increase the stability of the produced nanoparticles. This time there are two contributing factors for nanoparticle formation. In addition to the desolvating factor of EtOH to protein, now the aqueous protein solution is desolvating celastrol due to its low solubility in water. We were expecting celastrol to aggregate due to the addition of water and further interact with the exposed hydrophobic residues of HSA burying the lipophilic cargo into a polar shell. If celastrol and protein did not interact, we would expect to see two populations of nanoparticles, which would likely be evident in the size distributions obtained with DLS. Furthermore, amorphous protein and crystalline celastrol would have different morphologies. Similar to BSA‐based nanoparticles, the EtOH to protein solution ratio was investigated for HSA/celastrol co‐nanoparticles (Figure 4 b). Even though the size distribution (Figure 4 a) was slightly broader than in the case of HSA alone, only one population was achieved, which suggests an interaction between HSA and celastrol. A 4:1 flow rate ratio gave a distribution with an average size of 105.0±0.7 nm (PDI=0.026) for HSA and 122.5±0.9 nm (PDI=0.107) for HSA with celastrol (Figure 4 a), which are ideal for drug delivery purposes.^1^ Furthermore, similarly to the BSA‐based nanoparticles (Figures 2 c and 2 f), the produced HSA/celastrol particles were spherical as determined from the TEM images (Figure 4 d). However, the higher contrast in the TEM images suggests higher density of the nanoparticles than in case of pure BSA nanoparticles. This could be due to the addition of celastrol inside the nanoparticle matrix. To quantify the amount of encapsulated celastrol and get an estimate of the encapsulation efficiency (EE%) HPLC was used. An EE% of 75±24 % was achieved by comparing the amount of celastrol injected in the co‐flow device to the amount free in solution after nanoparticle formation.

**Figure 4:**
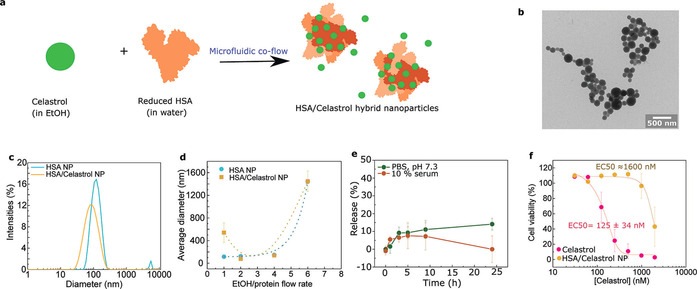
Production of HSA/celastrol hybrid nanoparticles. (a) Celastrol is encapsulated within HSA nanoparticles by the microfluidic co‐flow device. This was achieved by adding celastrol to the EtOH phase and HSA as the protein phase. (b) TEM images of HSA/celastrol nanoparticles formed by using a 4:1 flow rate ratio (EtOH/protein). (**c**) The resulting hybrid nanoparticles have slightly broader size distributions. (d) The average diameter sizes were comparable to those of pure HSA nanoparticles. (e) Stability of HSA/celastrol nanoparticles in PBS and in 10 % human serum, followed over a 24 h time period. The release of celastrol from the nanoparticles to the outside environment was determined by HPLC. (f) Cell viability in RAW 264.7 murine macrophages with different concentrations of celastrol, either free in solution or incorporated into HSA nanoparticles.

In addition to encapsulation, the release kinetics of the cargo molecules is important for drug delivery applications. The release should not happen before the nanoparticle has reached its target. The release profiles of celastrol were followed by using HPLC. Two experiments were conducted: in the first, the nanoparticles were placed in PBS (pH 7.3) and in the second experiment, the nanoparticles were mixed with 10 % human serum for 24 h (Figure 4 e). The release kinetics for both samples follows the same trend over a 10 h period reaching ≈10 % release, but after 24 h the celastrol concentration in the solution drops to 0 in the sample containing human serum. This could mean either that the celastrol concentration is too high and it forms aggregates, which are then not detected, or that celastrol is not stable in human serum. Finally, the cell toxicity of free celastrol and HSA/celastrol nanoparticles were examined (Figure 4 f) and show high toxicity with free celastrol with an EC_50_ of 125±34 nm. However, when celastrol is encapsulated within HSA nanoparticles its toxicity is greatly reduced (EC_50_ approximately 1600 nm). Indeed, using encapsulated celastrol, a dose 10 times higher can be tolerated by cells. Cell toxicity is an important consideration for biomedical applications and should be minimized in order to reduce potential side effects that occur as a result off target interactions with healthy cells. Even with the best available targeting strategies only a portion of the injected drug molecules end up in the target tissue. Thus, reducing the cytotoxicity of a drug significantly decreases potential side effects.

Protein nanoparticles have gained considerable attention owing to their high binding capacity of various drugs and low immunogenic response, which minimises adverse side‐effects. Herein, a microfluidic platform for generating protein nanoparticles for drug delivery was presented. A microfluidic co‐flow method was established in which sub‐micrometre‐sized protein particles were created. By using this co‐flow method, HSA nanoparticles were formed with homogeneous size distribution and we showed that by varying the flow rates of the different components, the average size of the nanoparticles can be modulated from ≈100 nm to 1 μm. The nanoparticles were stabilized by intermolecular disulfide bonds by reducing HSA prior to co‐flowing and by giving them time to re‐oxidise after the co‐flow, which eliminates the need for toxic crosslinkers. We further demonstrate that highly lipophilic celastrol can be encapsulated into our nanoparticles, which increases its solubility in aqueous solutions and decreases its cell toxicity. Future work will determine the potential of these particles for targeted drug delivery.

## Conflict of interest

The authors declare no conflict of interest.

## Supporting information

As a service to our authors and readers, this journal provides supporting information supplied by the authors. Such materials are peer reviewed and may be re‐organized for online delivery, but are not copy‐edited or typeset. Technical support issues arising from supporting information (other than missing files) should be addressed to the authors.

Supplementary
