# Supplementary material for: A Microfluidic Co‐Flow Route for Human Serum Albumin‐Drug–Nanoparticle Assembly
Source: Chemistry. 2020 Apr 28;26(27):5965–9. doi: 10.1002/chem.202001146 (PMC7318336; doi:10.1002/chem.202001146)
Supplement: Supplementary file 1 — Supplementary [file CHEM-26-5965-s001.pdf]

Chemistry–A European Journal

Supporting Information

A Microfluidic Co-Flow Route for Human Serum Albumin-Drug–Nanoparticle Assembly

Tuuli A. Hakala,[a] Sarah Davies,[a] Zenon Toprakcioglu,[a] Barbara Bernardim,[a] Gonçalo J. L. Bernardes,*[a, b] and Tuomas P. J. Knowles*[a]

Experimental Procedures

Fabrication of microfluidic chip

3D co-flow device was designed with AutoCAD (Autodesk) and made by combining standard photolithography and soft lithography techniques. Two different masters were fabricated and resulted in PDMS structures aligned together to achieve 3D-channel structure in which fluids flow next to each other.

The device was made with standard photolithography methods by using SU8 (MicroChem) 3025 or 3050 depending on the desired height.Two different master molulds were fabricated to create 3D co-flow device (Fig SI1). First mask consisted from three different layers (Fig SI 1a) and the second from two (Fig SI 1b). Fist, 25 µm layer of SU8 3025 photoresist was spun onto a silicon wafer at 3000 RPM and left to bake at 95 ºC for 15min. Next, the wafer was exposed with UV light through a photomask (MicroLithography Services) with a negative image of the protein channel design followed by 2 min post-exposure bake in 95 ºC. Next, a second layer of 25 µm was spun on the wafer and baking and UV exposure steps were repeated. This time mask with ethanol inlet and channel was used. The third and final layer of 25 µm SU8 was applied to result total thickness of 75 µm. This layer was left to bake for 45 min before UV exposure through a mask with water inlet and co-flow channel. After post exposure bake of 5 min the master mould was developedin propylene glycol mono methyl ether acetate (Sigma) until uncrosslinked SU8 was removed and the channel structure was revieled.. The second master mould was fabricated otherwise with same recipe, but the first layer was left out and the thus 50 µm SU8 layer was spun for the ethanol channel.

These masters were used as moulds in a soft lithography process[1] to fabricate the actual devices. Shortly, PDMS monomer and crosslinker (Sylgard 184, DowCorning) were mixed together in 10:1 ratio This mixture was degassed and baked at least for an hour at 65 ºC. Next, PDMS was cut and peeled off the master and inlets punched. To create the 3D structures, both PDMS layers were exposed to 40 W O_2 plasma for 30 s (Diener electronics) and aligned together. A small drop of ethanol was used to wet the surface prior to alignment to make sure the layers would not bind before a good alignment was achieved. The bonding was finalized at 65 ºC for at least 1.5 h. Finally, the chips were bonded to a glass slide for support (O_2 plasma, 15 s, 40 W).

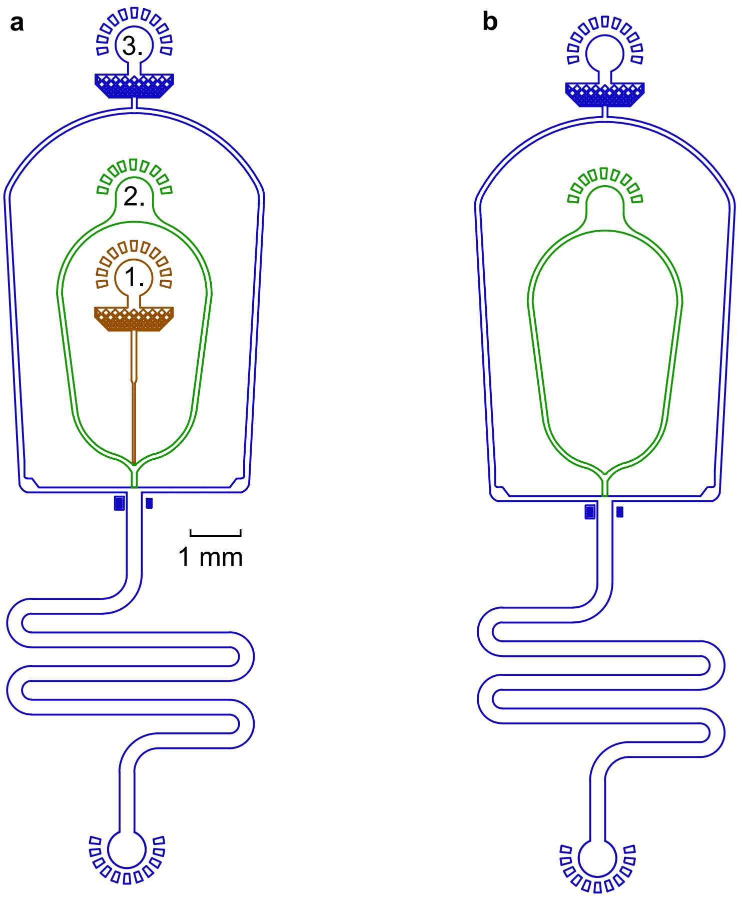

Figure S1. CAD design of the co-flow device masters. a) The first master was fabricated from three layers. First the protein inlet (1, 25 µm height), second the EtOH inlet (2, 50 µm height) and finally the water inlet (3, 75 µm height). b) second master was otherwise identical, the inner most channel (protein channel) was left out. When the PDMS channels prepared with these two master moulds were aligned and bonded resulting heights were: 25 µm protein channel, 100 µm EtOH channel and 150 µm water channel.

Nanoparticle Formation by Co-flow
Protein (BSA or rHSA) was reduced with glutathione (GSH) by using a known protocol.[2,3] Protein solution in water was incubated with GSH (200 equivalents) for 2 h at 37 ºC prior to nanoparticle formation. Excess GSH was removed by overnight dialysis with water at 4 ºC or by using a centrifuge filter (Amicon filter).

Protein was adjusted to appropriate concentration and co-flow in the microfluidic device with EtOH and milliQ water. Protein was introduced with the innermost inlet (1. In figure SI 1a), EtOH from the middle inlet (2. In figure SI1a) and milliQ water from the outer most inlet (3. In figure SI1 a).MilliQ was used as a carrier fluid to make sure the nanoparticles form away from hydrophobic PDMS surface and limits the material sticking to the PDMS surface. The formed nanoparticles were collected from the outlet of the device and the oxidation of intramolecular disulfide bonds was finalized at 37 ºC. EtOH was washed away and nanoparticles moved into buffer by overnight dialysis at 4 ºC in PBS.

To produce HAS/Celastrol nanoparticles reduced HSA solution (5 mg/ml) was co-flowed with celastrol in EtOH and MilliQ in a microfluidic 3D device. 5:1 w:w (HSA:celastrol) ratio was used with all celastrol encapsulation experiments. Otherwise the protocol followed the steps for protein nanoparticle preparation.

Synthesis of (E)-N-ethyl-4-oxo-4-phenylbut-2-enamide was prepared by a modified reported procedure.[4] 1.12 g (6.4 mmol) of commercially available 3-benzoylacrylic acid were dissolved in dimethoxyethane (13 mL) and cooled to –10 ºC under argon. Isobutylchloroformate (1.18 mL, 7.7 mmol) was added followed by 0.85 mL (7.7 mmol) of *N*-methylmorpholine. Afterwards, 8.0 ml (16.0 mmol) of a 2M solution of ethylamine in THF was added and stirred for 15 min at –10 ºC and 1 h at room temperature. 10 mL H_2O was added and the mixture was extracted with CH_2Cl_2 (3 x 15 mL). Organic layers were combined, washed with 10 mL sat. aqueous NaOH solution and H_2O, dried over anhydrous Na_2SO_4 and evaporated. The residue was purified by flash chromatography (10 – 80% EtOAc/hexanes) to yield pure *(E)*-*N*-ethyl-4-oxo-4-phenylbut-2-enamide (1.04 g, 5.10 mmol, 80% yield) as a pale yellow solid: R_f = 0.47 (80% EtOAc:hexanes); ^{1}H NMR (400 MHz, Chloroform-*d*) 8.05 – 8.01 (m, 2H), 7.97 (d, *J* = 15.0 Hz, 1H), 7.64 – 7.57 (m, 1H), 7.54 – 7.47 (m, 2H), 6.95 (d, *J* = 15.0 Hz, 1H), 5.97 (s, 1H), 3.45 (qd, *J* = 7.3, 5.7 Hz, 2H), 1.23 (t, *J* = 7.3 Hz, 3H); ^{13}C NMR (100 MHz, CD_3CN) 191.1, 164.5, 138.0, 136.9, 134.5, 133.1, 129.8, 129.6, 35.2, 14.7; HRMS ESI$^+$ (*m/z*): Calcd. For $C_{12}H_{14}NO_2^+$ [M + H]$^+$ 204.1019, found 204.0995; FTIR (cm^{-1}): 3258, 1637, 1593, 1559, 1447, 1357, 1324, 1289, 1196, 1009, 977. ^{1}H NMR and ^{13}C NMR spectrum are presented in **Figure S2** and **S3**.

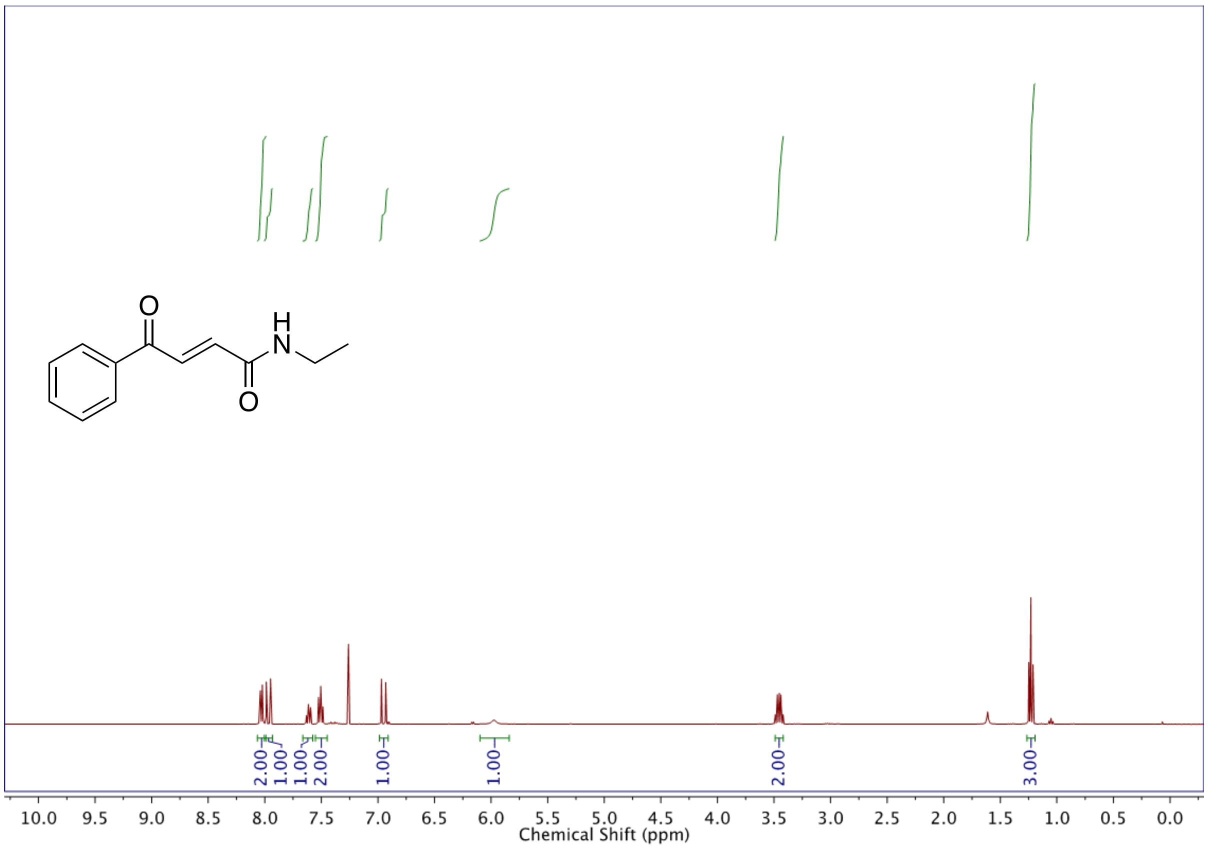

Figure S2. ^{1}H NMR spectrum of (*E*)-*N*-ethyl-4-oxo-4-phenylbut-2 enamide.

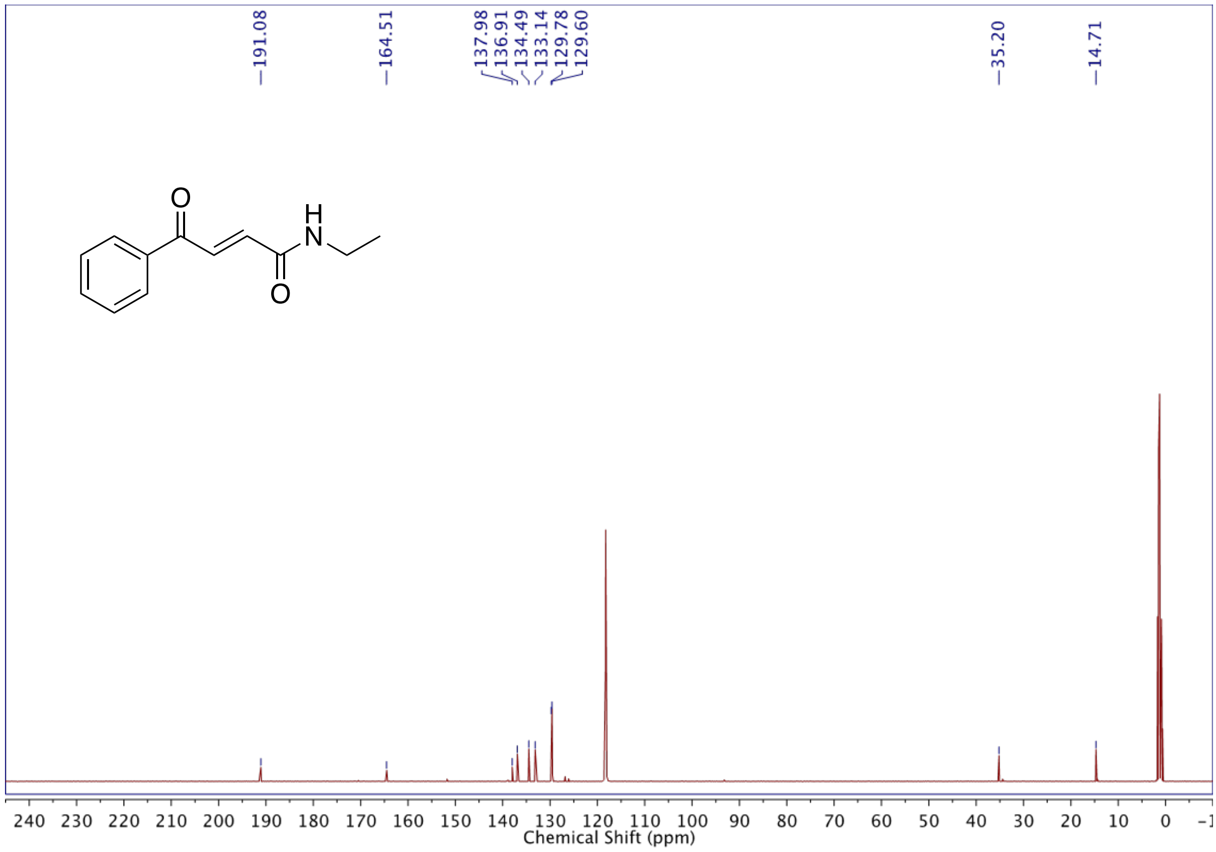

Figure S3. 13C NMR spectrum of (*E*)-*N*-ethyl-4-oxo-4-phenylbut-2 enamide.

Blocking free cysteine in BSA with carbonylacrylic acid reagent

Cysteine specific (*E*)-*N*-ethyl-4-oxo-4-phenylbut-2-enamide (**caa**) reagent was used to block free cysteine residues on BSA. The reagent (5 equivalents, excess) was dissolved in dimethyl sulfoxide and added to the BSA solution in 50 mM phosphate buffer (pH 8, pH 7.4). The reaction was incubated for 1 h in 37 ºC and the uncreated **caa** was removed with 10 kDa cut off spin filter. LCMS was used to confirm the conjugation (**Figures S4** and **S5**). The LCMS data was acquired Waters SQ detector 2 mass spectrometer. This was coupled with an Acquity UPLC system equipped with Acquity Q6 UPLC Protein BEH C4 column (300 Å, 1.7 μm, 2.1mm x 50 mm). The mobile phase at flow fate of 0.2 mL/min composed from solvents; A water with 0.1% formic acid and B 71% acetonitrile, 29% water and 0,075% formic acid. Gradient was programmed from 28% B to 71.2% B in 12 min followed by 1 min at 71.2% B, 100% N for 2 and 28% A for 2 min. Electrospray was conducted with capillary voltage of 3.0 kV and cone voltage of 30 V. Desolvating nitrogen gas flow was kept at 800 L/h through the run. The data was recustructed and analysed using MassLynx software (version 4.1, Waters) according to manufacturer's instructions.

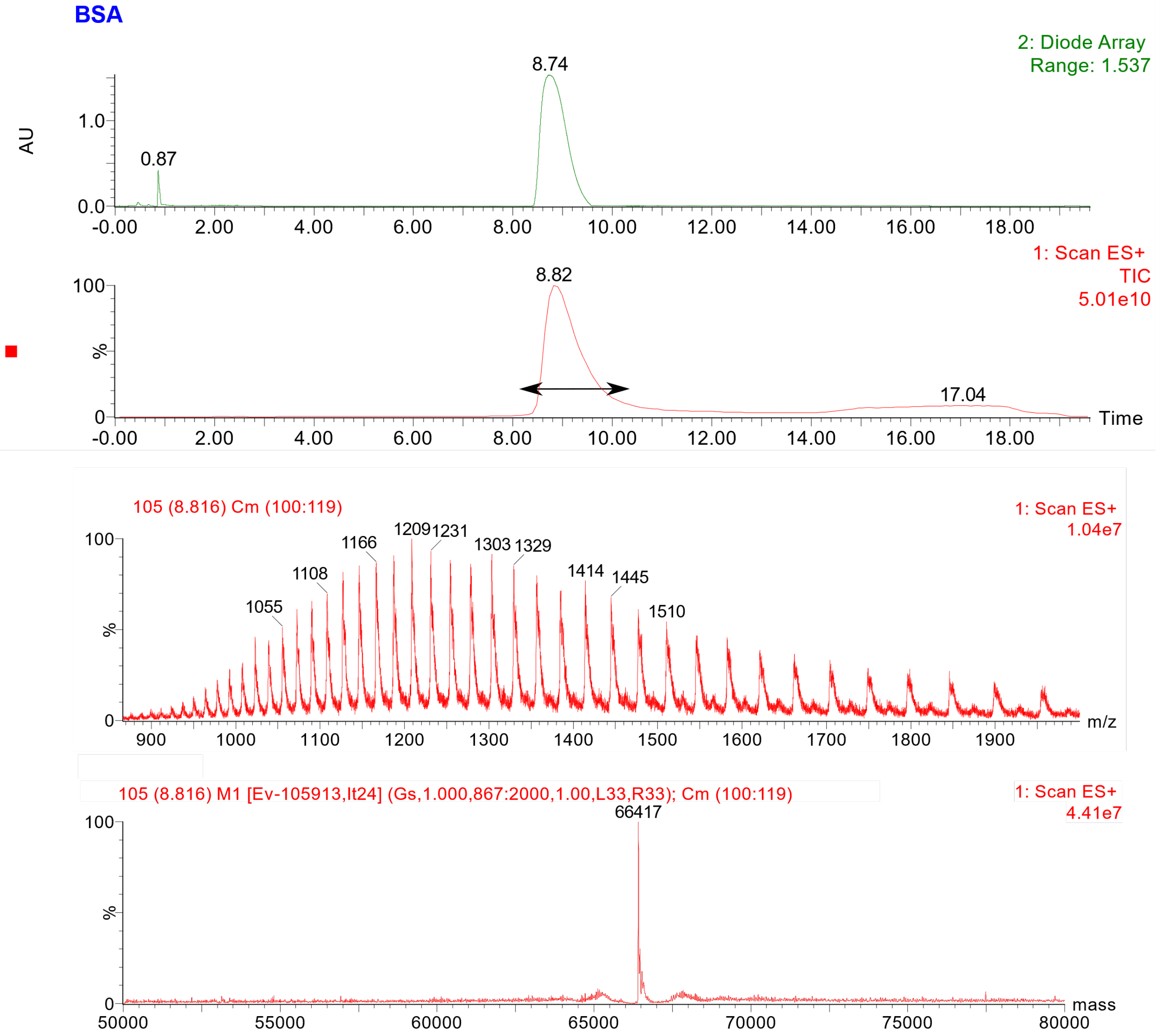

Figure S4. LCMS spectrum of BSA.

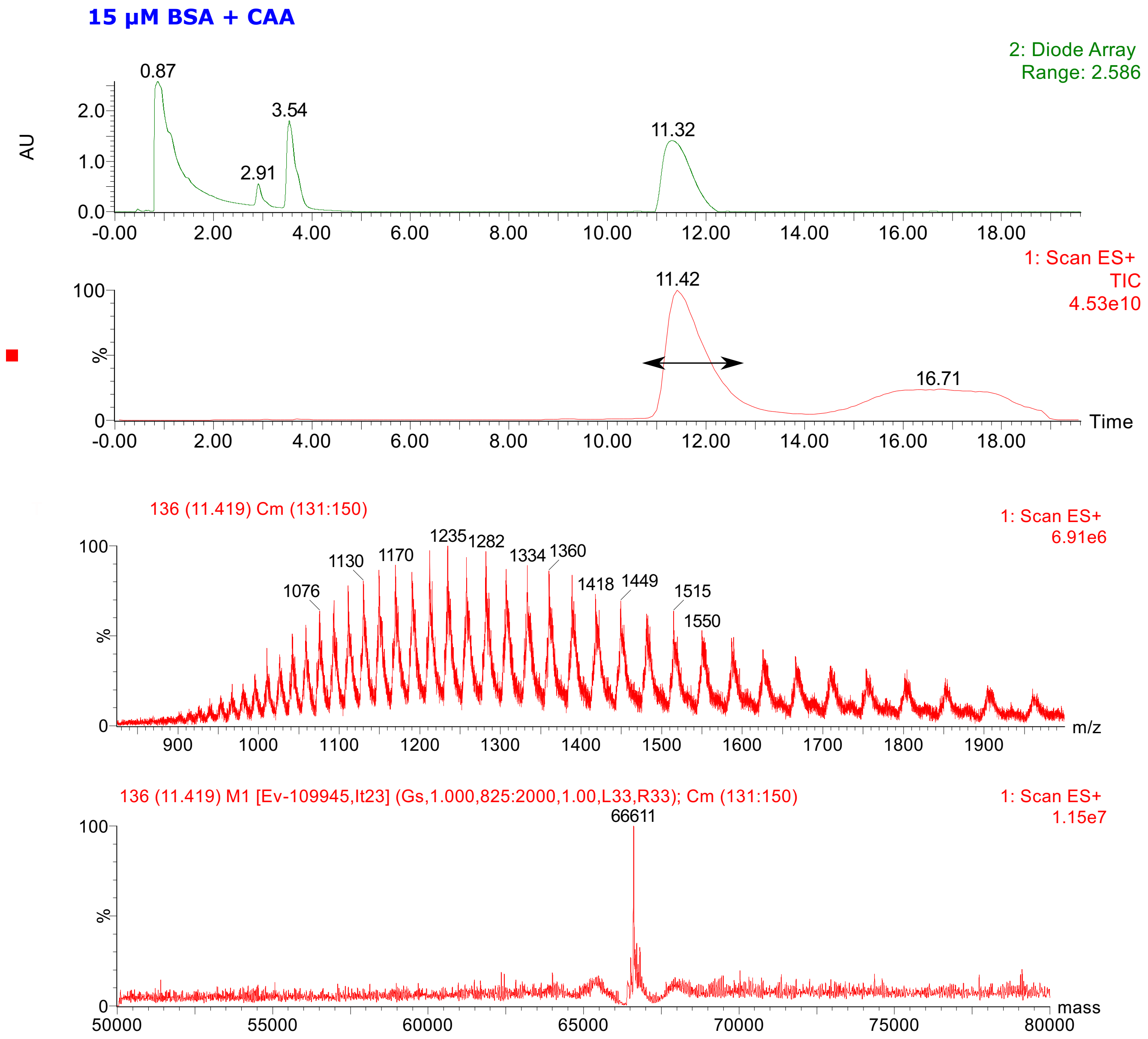

Figure S5. LCMS spectrum of BSA+caa.

Dynamic light scattering (DLS) and zeta potential experiments
DLS and zeta potential experiments were conducted with a Malvern Zeta Sizer. DLS samples were loaded in to a low-volume disposable cuvette (Brand® 759200). The cuvette was loaded with 70 μL sample and size distribution was calculated as an average of three sequential measurements. Zeta potential measurements were performed with disposable folded capillary cell (DTS1070 from Malvern panalytical) using diffusion barrier method. First, the cuvette was filled with PBS, followed by carefull injection of 100 µl sample volume into the bottom of the folded capillary cell. Finally, the zeta potentials were recorded using Smoluchowski equation.

Table S1. The average diameter and polydispersity index (PDI) was measured for each nanoparticle condition used. Each condition was measured with at least two different chips.

Sample	EtHO/Protein flow rate	Average diameter (nm)	PDI
BSA NP (1 mg/ml BSA)	0.2	181 ± 44	0.281
	0.50	159 ± 7.5	0.176
	1.00	192 ± 7.3	0.2
	3.00	300 ± 16	0.137
	5.00	862 ± 56	0.14
BSA NP (10 mg/ml BSA)	0.2	123 ± 1.9	0.386
	0.50	145 ± 4.7	0.505
	1.00	320 ± 3.6	0.322
	3.00	393 ± 3.0	0.507
	5.00	534 ± 7.8	0.541
HSA NP	1.00	111 ± 6.9	0.205
	2.00	106 ± 21	0.262
	4.00	129 ± 22	0.052
	6.00	1340 ± 165	0.2
HSA/Celasrol NP	1.00	540 ± 170	0.207
	2.00	76.0 ± 1.7	0.142
	4.00	139 ± 2.8	0.236
	6.00	1450 ± 180	0.355

Transmission electron microscopy sample preparation and imaging
The sample was either sprayed straight onto the TEM grid or deposited by pipette (aqueous samples). Sample deposition was followed by staining with 2% uranyl acetate (1 min) and washing with MilliQ water (2 x 10 μL). TEM images were recorded at the Cambridge Advanced Imaging Centre with Tecnai G2 electron microscope.

Surface hydrophobicity measurement
Surface hydrophobicity was measured by using ANS (8-Anilino-1-naphthalenesulfonic acid). BSA nanoparticles were produced as aforementioned using 1:1 flow ratio. BSA and BSA nanoparticles were prepared in PBS (pH 7.2) and adjusted to 3 µM concentration with 75 µM ASN. The solution was incubated for 15 min in the absence of light. The fluorescence spectra were recorded with a fluorescence spectrometer (Varian Cary Eclipse) using 388 nm excitation. Background measurement (ANS + buffer) was conducted and subtracted from the sample spectra.

Determination of celastrol concentration by HPLC
A calibration curve (**Figure 6**) for celastrol by HPLC was created by using KR100-5-C8 column and 1 mL/min flow of 15:85 of ammonium acetate (5 mM) with acetic acid (0.1%) and acetonitrile with acetic acid (0.1%). Emodin (75 µM) was used as an internal standard. Retention times were 3.8 min for celastrol and 2.3 min for Emodin (**Figure S7**).

The amount of encapsulated celastrol was determined by measuring the free celastrol in the solution after the formation of HSA-celastrol nanoparticles. This result was then compared to the celastrol concentration put in. The EE% was calculated with the equation:

$$EE\% = \frac{[Celastrol_{tot}] - [Celastrol_{free}]}{[Celastrol_{tot}]}, \quad (1)$$

in which $[Celastrol_{tot}]$ is the total celastrol added to the system and $[Celastrol_{free}]$ the free non-entrapped celastrol in NP solution.

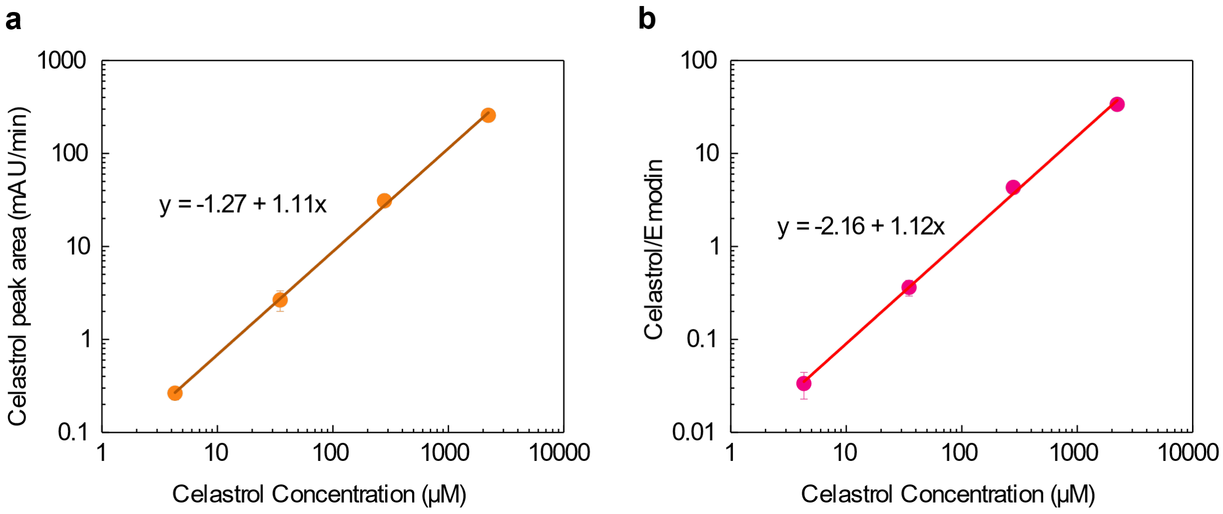

Figure S6. The celastrol concentration is linearly proportional to the peak area of celastrol and the celastrol/emodin peak relation in the HPLC analysis.

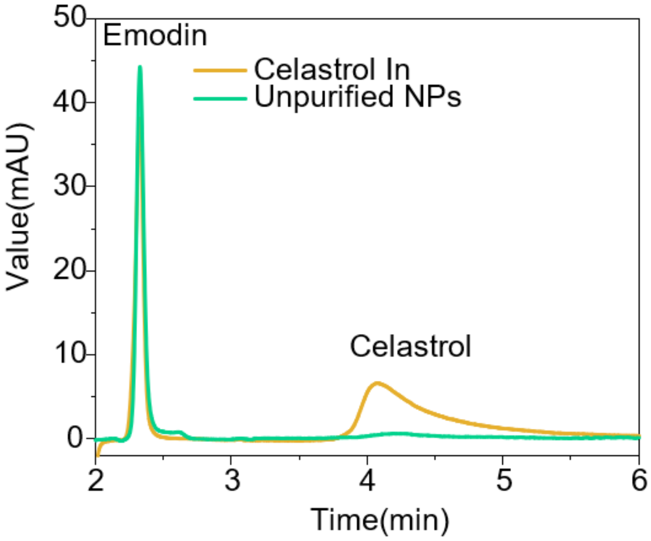

Figure S7. Determination of nanoparticle encapsulation efficiency with HPLC Chromatochram from celastrol (yellow) and supernatant from celastrol HSA nanoparticles (green).

Celastrol release kinetics with HPLC

Aliquots (100 µL) of nanoparticle solution in PBS were incubated at 37 ºC. At each time point cold acetonitrile (100 *µ*L) was added to aliquot to precipitate any protein and filtered through 0.22 µm syringe filter before analysis by HPLC (Thermo Fisher). The same HPLC conditions were used here as described above. The released celastrol was analysed by integrating the celastrol peak by using OriginLab and comparing it to the total amount of celastrol in the nanoparticle solution.

Cell culture

RAW264.7 (ATCC TIB-71) cells were routinely grown in a humidified incubator at 37 ºC under 5% CO_2, and split before reaching confluence using a cell scraper. The cell line was grown on DMEM medium supplemented with 10% heat-inactivated FBS, 4 mM L-glutamine, 4500 mg/L glucose, 1 mM sodium pyruvate, 1500 mg/L sodium bicarbonate, and 100 units/mL penicillin. All reagents were bought from Gibco, Life Technologies (USA), unless otherwise stated.

Cytotoxicity in RAW264.7 cells

Cytotoxicity of celastrol and celastrol encapsulated in nanoparticles was assessed by using a CellTiterBlue Cell Viability Assay (ThermoFisher Scientific, USA). Briefly, cells were seeded at a concentration of 5,000 cells/well (200 µL) in flat-bottom 96-well plates and allowed to adhere and adapt to the plates for 24 h. At this point, increasing concentrations of each compound (celastrol dissolved in EtOH, nanoparticles dissolved in PBS) were added in technical triplicates: 31, 63, 125, 250, and 500 nM. Final concentration of EtOH in each well was < 0.5%. Plates were incubated for 3 days, after which time cell viability was assessed by the addition of CellTiterBlue

Reagent (dilution 1:10 from commercial stock) and incubation for a further 3 h, before analysis of fluorescence on an Infinite M200 (Tecan, USA) plate-reader (excitation: 570 nm, emission: 590 nm).

References

[1] J. C. McDonald, G. M. Whitesides, *Acc. Chem. Res*. **2002**, *35*, 491–499.

[2] W. Wang, Y. Huang, S. Zhao, T. Shao, Y. Cheng. *ChemComm* **2013**, *49*, 2234–2236.

[3] S. Zhao, W. Wang, Y. Huang, Y. Fu, Y Cheng. *MedChemComm* **2014**, *5*, 1658–1663.

[4] B. Bernardim, P. M. S. D. Cal, M. J. Matos, B. L. Oliveira, N. Martínez-Sáez, I. S. Albuquerque, E. Perkins, F. Corzana, A. C. B. Burtoloso, G. Jiménez-Osés, G. J. L. Bernardes, *Nat. Commun.* **2016**, *7*, 13128.
